# Significantly lower antigenicity of incobotulinumtoxin than abo- or onabotulinumtoxin

**DOI:** 10.1007/s00415-022-11395-2

**Published:** 2022-10-05

**Authors:** Harald Hefter, Dietmar Rosenthal, Alexander Jansen, Raphaela Brauns, Beyza Ürer, Hans Bigalke, Hans-Peter Hartung, Sven G. Meuth, John-Ih Lee, Philipp Albrecht, Sara Samadzadeh

**Affiliations:** 1 Department of Neurology, Medical Faculty, Heinrich-Heine-University, Moorenstrasse 5, 40225 Düsseldorf, Germany; 2 Institute of Toxicology, Medical School Hannover, Hannover, Germany; 3 Department of Neurology, Palacky University Olomouc, Olomouc, Czech Republic; 4 Brain and Mind Center, University of Sydney, Sydney, Australia; 5 Department of Neurology, Medical University of Vienna, Vienna, Austria; 6 Experimental and Clinical Research Center, Charité-Universitätsmedizin Berlin, Freie Universität Berlin and Humboldt-Universität zu Berlin, Berlin, Germany

**Keywords:** Difference in antigenicity, Neutralizing antibodies, IncobotulinumtoxinA, Complex proteins, Botulinum toxin type A preparations

## Abstract

**Background:**

For many indications, BoNT/A is repetitively injected with the risk of developing neutralizing antibodies (NABs). Therefore, it is important to analyze whether there is a difference in antigenicity between the different licensed BoNT/A preparations.

**Methods:**

In this cross-sectional study, the prevalence of NABs was tested by means of the sensitive mouse hemidiaphragm assay (MHDA) in 645 patients. Patients were split into those having exclusively been treated with the complex protein-free incoBoNT/A preparation (CF-MON group) and those having started BoNT/A therapy with a complex protein-containing BoNT/A preparation (CC-I group). This CC-I group was split into those patients who remained either on abo- or onaBoNT/A (CC-MON group) and those who had been treated with at least two BoNT/A preparations (CC-SWI group). To balance treatment duration, only CC-MON patients who did not start their BoNT/A therapy more than 10 years before recruitment (CC-MON-10 group) were further analyzed. The log-rank test was used to compare the prevalence of NABs in the CF-MON and CC-MON-10 group.

**Results:**

In the CF-MON subgroup, no patient developed NABs. In the CC-I group, 84 patients were NAB-positive. NABs were found in 33.3% of those who switched preparations (CC-SWI) and in 5.9% of the CC-MON-10 group. Kaplan–Meier curves for remaining NAB-negative under continuous BoNT/A therapy were significantly different (*p* < 0.035) between the CF-MON and CC-MON-10 group.

**Conclusion:**

Frequent injections of a complex protein-containing BoNT/A preparation are associated with significantly higher risks of developing NABs than injections with the same frequency using the complex protein-free incoBoNT/A preparation.

## Introduction

The popularity of the use of botulinum neurotoxin type A (BoNT/A) is rapidly increasing. In dermatology, BoNT/A injections have become the most popular of all cosmetic procedures worldwide [1, 2]. In neurology, the number of indications for BoNT/A treatment is continuously growing [3]. Additionally, in ophthalmology, urology, internal medicine, and orthopedics, BoNT/A is increasingly applied [1].

BoNT/A is a 150 kDa large molecule that is usually embedded in a 750 kDa large protein complex. Antibodies (ABs) can be induced against various parts of this BoNT/A complex. Some ABs do not reduce the efficacy [4], but some ABs targeting special epitopes of the 150 kDa large botulinum neurotoxin molecule [5, 6] are neutralizing antibodies (NABs) resulting in the development of partial (PSTF) or complete (CSTF) secondary treatment failure (STF). Because of the negative impact of NABs on clinical outcome [7, 8], the antigenicity of BoNT/A preparations has become an increasingly clinically relevant problem, especially with an increasing number of indications.

However, for a variety of indications, repetitive injections have to be performed to maintain a certain level of benefit of BoNT/A therapy [9]. This increases the risk of developing immune resistance against the botulinum toxin molecule [4]. Several risk factors for the induction of NABs have previously been suggested, such as booster injections and short interinjection intervals, high dose per session, BoNT/A preparation, and the duration of treatment [10, 11]. However, when a patient receives repetitive BoNT/A injections for multiple indications (e.g., to treat migraine and dystonia or spasticity of extremities and bladder dysfunction), the risk of developing NABs against BoNT/A may become markedly enhanced due to the increase in dose and the shortening of interinjection intervals due to various injections applied to the individual patient [11].

Both onabotulinumtoxinA (onaBoNT/A; Botox^®^, Allergan, USA) and abobotulinumtoxinA (aboBoNT/A; Dysport^®^, Ipsen, France) preparations contain proteins of the entire botulinum toxin type A complex built-up out of the botulinum neurotoxin molecule as well as out of hemagglutinins and non-hemagglutinin proteins [12]. These proteins are necessary for the passage of the acidic milieu of the stomach and penetration through the gastrointestinal wall [1, 13]. However, these bacterial proteins are not needed for the muscle-relaxing action when BoNT/A is directly injected into the indicated muscles. In contrast, these complex proteins may stimulate the immune system [14] and act as adjuvant substances and may be more a hindrance than a help regarding the efficacy of botulinum toxin [14].

Soon after the broad clinical use of onaBoNT/A, it was realized that NABs occurred in a high percentage (> 30%) of continuously treated patients [4, 10]. This observation led to a modification in the manufacturing process, further purification, and a reduction in the protein load in the onaBoNT/A preparation from 25 ng/vial [15] to 5 ng/vial. [16] This resulted in an approximately sixfold decreased rate of antibody formation [16] and a reduction in the occurrence of STF [17].

The protein load of the more recently (in 2005) licensed incobotulinumtoxinA (incoBoNT/A; Xeomin^®^, Merz Pharmaceuticals, Germany) is even lower (0.44 ng/vial [12]) than the protein load of the “new” onaBoNT/A and aboBoNT/A (4.35 ng/vial; [18]), suggesting an even lower antigenicity of incoBoNT/A than ona- and aboBoNT/A. During the manufacturing and purification process of incoBoNT/A, the complex proteins as well as inactive fragments of the BoNT/A molecule are removed [12]. This considerable reduction in the protein content and the resulting low antigenicity of incoBoNT/A has been shown in an animal experiment [19].

A comparative clinical trial has not been conducted, and consequently, a significant difference in the antigenicity of the three different BoNT/A preparations has not yet been demonstrated in clinical trials [11]. The low antibody rates published to date estimate the incidence of NAB induction rather than long-term prevalence because of the relatively short duration of the studies. However, these NAB incidences sum up to a rather high prevalence of more than 15–20% after 10–20 years of continuous BoNT/A treatment [11, 20, 21].

Therefore, in this cross-sectional comparative study, we analyzed the prevalence of NABs in 645 patients and compared the antigenicity of the complex protein-free incoBoNT/A preparation with that of complex protein-containing BoNT/A preparations in patients who were treated over a period of time of up to 10 years.

## Methods

This study was performed according to the guidelines of good clinical practice (GCP) and according to the Declaration of Helsinki. It has been approved by the local ethics committee of the University of Duesseldorf (number: 4085).

### Patients

All patients (*n* > 1250) in the BoNT ambulance of the Department of Neurology of the University of Düsseldorf (Germany) were informed of the aims of the study. Patients who fulfilled the inclusion criteria and gave written informed consent (see below) were consecutively recruited.

The inclusions were as follows: (i) adult patients with a movement disorder and (ii) patients under current treatment in the botulinum toxin department of the University of Düsseldorf. Exclusion criteria were: (i) patients under legal care, (ii) patients under current psychiatric treatment, and (iii) patients who missed more than 2- of the 3-month injection cycles.

The entire cohort was split into four different disease entity groups [FD = facial dystonia (hemifacial spasms or blepharospasm), CD = cervical dystonia, ODT = other dystonia (Meige syndrome or oromandibular or oropharyngeal dystonia), and SPAS = patients with spasticity].

### Study design and treatment

This monocentric, cross-sectional study took place at the Department of Neurology of Heinrich Heine University, Düsseldorf, Germany. Recruitment lasted from 1/2015 until 6/2017. Finally, 645 patients were recruited. An interim analysis was performed in 2016 and published 1/2019 [11].

Patients were recruited during their regular outpatient clinic visit to receive their next routine 3-months BoNT/A injection. Patients underwent a detailed neurological investigation, and a blood sample was taken for the determination of antibodies before the routine injection was performed.

Blood samples were centrifuged, deep-frozen, and stored until the interim analysis date was reached or recruitment had been finished. Patient personal and treatment-related data, including the indication for BoNT/A therapy, were collected from the charts. For the sake of comparison, the dose per session was transformed and unified by leaving ona- and incoBoNT/A doses unchanged and dividing aboBoNT/A doses by 3. This 1:3 conversion ratio was used in a previous study [20] and is in line with the European Consensus document, which suggests a conversion ratio of 1 U:3 U or 1 U:4 U between inco- and aboBoNT/A [22].

**Fig. 1:**
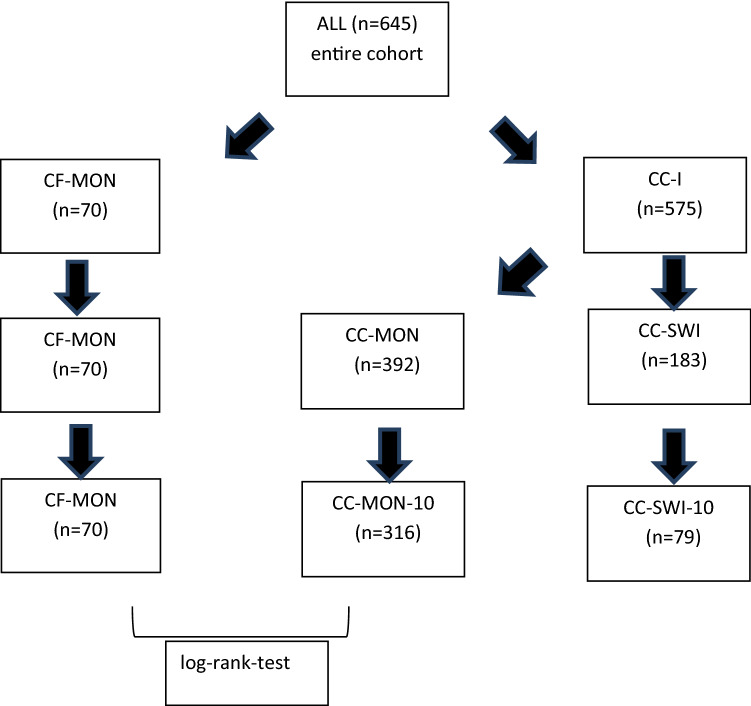
Diagram demonstrating the stratification process of treatment groups for final statistical comparison

For adequate statistical analysis, the entire cohort (ALL) was split into six different treatment groups of patients (see tables and Fig. 1). The complex protein-free monotherapy group (CF-MON group) comprised all patients who had exclusively been treated with the complex protein-free preparation incoBoNT/A, and the complex protein-containing group (CC-I group) contained all patients who had initially been treated with the complex protein-containing abo- or onaBoNT/A preparation. The CC-I group was split into those patients who remained on either abo- or onaBoNT/A during their entire BoNT/A therapy (CC-MON group) and into a group of patients who had been switched to another BoNT/A preparation because of a diminished clinical response to abo- or onaBoNT/A (CC-SWI group). Since in Germany incoBoNT/A was licensed in 2005, while abo- and onaBoNT/A were licensed in 1990, the duration of treatment had to be balanced among the test groups. Therefore, the analysis was restricted to patients in the CC-MON and CC-SWI group in whom BoNT/A therapy had not been initiated more than 10 years before recruitment (CC-MON-10 group and CC-SWI-10 group).

### Antibody testing

After collection of the clinical data, blood samples were coded by a running number and tested for the presence of ABs with an ELISA screening test by an independent, blinded contractor (BSL Bioservice Scientific Laboratories, Planegg, Germany). ELISA-positive samples were sent to another blinded contractor (Toxogen^®^ GmbH, Hannover, Germany) for the detection of NABs by means of the mouse hemidiaphragm assay (MHDA). The outcome measure of MHDA is paralysis time [23]. When the paralysis time of a sample was longer than 60 min, the sample was classified as NAB-positive. After all samples had been analyzed, a list of the paralysis times for all samples and whether they were MHDA-positive was returned to our institution.

### Statistical analysis

A chi^2^ test was used for the female/male NAB prevalence comparison. A t test with unequal variances was used to compare the duration of treatment and the unified dose per session on the day of recruitment in the CF-MON and CC-MON-10 groups. A Kaplan–Meier analysis (KMA) and the corresponding analysis of the number of patients at risk were performed for all treatment groups to determine the probability of remaining NAB-negative during BoNT/A therapy. A statistical comparison of the KMAs using the log-rank test was performed only between the complex protein-free (CF-MON) and the complex protein-containing (CC-MON-10) groups, testing the hypothesis that the probability of remaining NAB-negative during BoNT/A treatment depended on the BoNT/A preparation used. All statistical tests were performed using the SPSS^®^ statistics package (version 25; IBM, Armonk, USA).

#### Role of the funding source

The private non-profit institution “Inge-Diesbach Stiftung” has interest to support the research work of HH and his team. This funding did not have any influence on the design, the performance, and presentation of the study.

## Results

### Demographic and treatment-related data and distribution of disease entities in the entire cohort and six different treatment subgroups

A total of 645 patients (ALL group) were enrolled; 395 patients were females and 250 were males. The mean duration of treatment was 2885 days (7.90 years). The mean dose (at the recruitment session) was 210 uDU (Table 1).

**Table 1:** Demographic and treatment-related data in the different treatment groups

Group	*N* =	Age (years)	Sex f/m	N = / % AK +	Incidence (AK + /year)	Duration (days) MV/SD	Dose (uDU) MV/SD
ALL	645	61.3/13.0	395/250	84/13.0	1.65	2885/1966	210/118
CF-MON	70	55.3/13.3	37/33	0/0.0	0.0	1715/1106	265/110
CC-I	575	62.0/12.7	358/217	84/14.6	1.77	3019/1999	203/116
CC-MON	392	62.0/13.0	238/157	23/5.9	0.84	2546/1603	178/112
CC-SWI	183	62.0/11.4	120/63	61/33.3	2.71	4048/2362	257/105
CC-MON-10	316	61.2/13.6	204/116	19/6.0	1.13	1938/963	191/107
CC-SWI-10	79	59.8/11.5	56/23	20/25.3	5.06	1828/935	210/109

*N* number of patients, *f* female, *m* male, *AK+* number of patients with a positive MHDA test, *%AK+* percentage of MHDA-positive patients in a group, *MV* mean value, *SD* standard deviation, *uDU* unified dose unit (see “Methods”); for the definition of the treatment groups, see “Methods”

The entire cohort was split into four different disease entity groups (FD, CD, ODT, and SPAS) (for details, see “Methods” and tables). The majority of patients suffered from CD (69.1%). The mean duration of treatment was the longest at 3214 days (8.8 years) in the FD group and approximately 2850 days (7.8 years) in the other three groups (see Table 2). The mean dose was the lowest in the FD group and the highest in the SPAS group (Table 2).

**Table 2:** Treatment-related data from the different disease entity groups and percentages of disease entities in the different treatment groups

	FD	CD	ODT	SPAS
*N* =	105	444	58	36
%AK + (%)	2.9	14.8	15.5	14.3
Incidence (%AK + /year)	0.33	1.93	1.98	1.81
Duration (days) MV/SD	3214/1802	2800/2045	2864/1706	2877/1794
Unified dose (uDU) MV/SD	40/44	249/85	145/118	328/100
	%/group	%/group	%/group	%/group
ALL	16.3	69.1	9.0	5.6
CF-MON	10.0	82.9	5.7	1.4
CC-I	17.1	67.4	9.4	5.9
CC-MON	22.7	58.9	10.7	7.7
CC-SWI	4.9	85.7	6.6	2.2
CC-MON-10	17.2	66.2	9.7	6.9
CC-SWI-10	7.7	83.3	6.4	1.4

*FD* patients with facial dystonia (see “Methods”), *CD* patients with cervical dystonia, *ODT* patients with other dystonia (see “Methods”), *SPAS* patients with spasticity, *N* number of patients, *%AK +* percentage of patients with a positive MHDA test, *MV* mean value, *SD* standard deviation, *uDU* unified dose units (see “Methods”); for the definition of the different treatment groups, see “Methods”

The entire cohort (ALL group) was split into 6 different treatment groups (Fig. 1, Tables 1, 2). The CF-MON group contained only 70 patients who had exclusively been treated with incoBoNT/A, with the highest mean dose per session on the day of recruitment of 265 uDU and the shortest mean duration of treatment (1715 days = 4.70 years). The CC-I group contained 575 patients who had started their BoNT/A treatment with either abo- or onaBoNT/A and were treated for a much longer time period (3019 days = 8.27 years) than the patients in the CF-MON group. Those 183 patients who had been switched to another BoNT/A preparation (CC-SWI group) were treated for the longest mean duration (4048 days = 11.09 years) with a fairly high dose per session (257 uDU). When they were removed from the CC-I group, 392 patients remained who were exclusively treated either with abo- or ona-BoNT/A (CC-MON group) with a mean duration of 2546 days (= 6.98 years) and the lowest mean dose (178 uDU). For the final analysis, those patients were selected) who did not start their BoNT/A 10 years before recruitment (CC-MON-10 and CC-SWI-10 groups).

The CF-MON and the CC-MON-10 groups were compared. The mean duration in the CF-MON group did not significantly differ (*p* = 0.108, n.s.) from that in the CC-MON-10 group (1938 days = 5.31 years), but the mean dose per session was significantly higher (*p* < 0.001) in the CF-MON group than in the CC-MON-10 group (see Table 1).

The distribution of indications was different for the different preparation subgroups. In the CC-MON-10 group, more patients were treated for facial dystonia (FD) than in the CF-MON group. The comparison of the CC-I group with the CC-MON and CC-SWI groups demonstrated that in the course of BoNT/A therapy, the BoNT/A preparation was switched in only a few FD patients, whereas in many CD patients, another BoNT/A preparation was used (Table 2).

### Prevalence and mean incidence of NABs in the entire cohort and the six treatment groups

Cross-sectional testing for antibodies in all 645 patients detected NABs in 84 patients (13%). The mean incidence of NAB induction was 1.65% per year (see Table 1). Overall, 53 of the 395 female patients (13.4%) and 31 of the 219 male patients (12.4%) were MHDA-positive. Chi^2^ testing did not show a significant difference in NAB induction between females and males (chi^2^ test: *p* > 0.41; n.s.).

The prevalence of NABs in the FD subgroup was less than 3%. In all other disease entity groups, the prevalence of NABs was higher than 14%. The mean incidence of NAB induction was approximately 0.33 in the FD subgroup and approximately 1.9% in all the other disease entity groups.

None of the patients who had exclusively been treated with the complex protein-free incoBoNT/A preparation (CF-MON group) tested positive in the MHDA.

NABs were detected in 61 out of 183 patients (= 1/3) in the CC-SWI group. In those 392 patients in the CC-MON group in whom BoNT/A preparation had not been switched and who remained on abo- or onaBoNT/A, only 23 (= 5.9%) patients tested positive in the MHDA. In the CC-MON-10 group with a duration of treatment comparable to that of the CF-MON group 19 (= 6%) patients were MHDA-positive.

### Kaplan–Meier analysis of the prevalence of NABs in the treatment groups

**Fig. 2:**
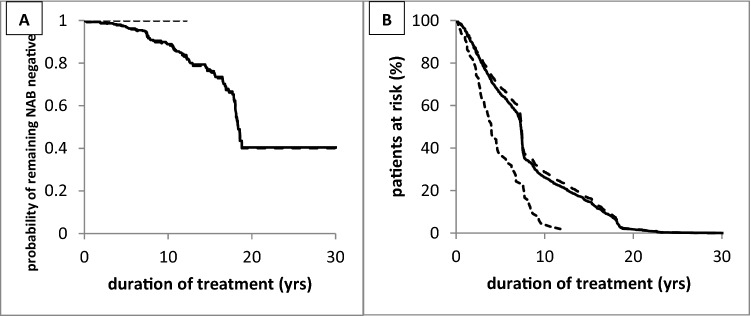
**A** Kaplan–Meier analysis of remaining MHDA-negative in the CF-MON group (light hatched line), ALL group (full line), and CC-I group (heavy hatched line). **B** Temporal development of the patients at risk in the CF-MON group (light hatched line), ALL group (full line), and CC-I group (heavy hatched line)

**Fig. 3:**
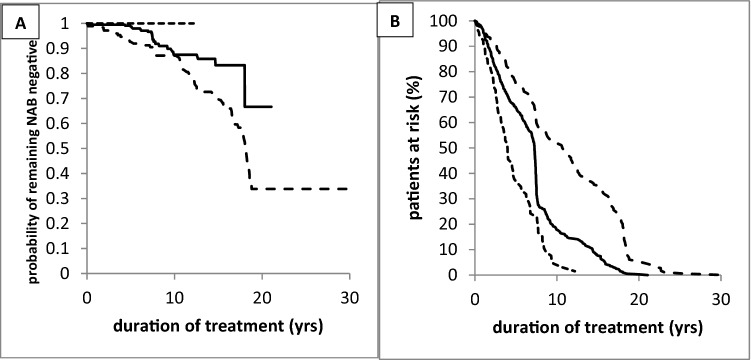
**A** Kaplan–Meier analysis of remaining MHDA-negative in the CF-MON group (light hatched line), CC-MON group (full line), and CC-SWI group (heavy hatched line). **B** Temporal development of the patients at risk in the CF-MON group (light hatched line), CC-MON group (full line), and CC-SWI group (heavy hatched line)

Since none of the patients in the CF-MON group had developed relevant NAB titers, the KMA curve of the probability of remaining NAB-negative is a straight line in Figs. 2A, B and 3A (hatched line). As a consequence, the KMA curves of the entire cohort (ALL group) and the CC-I group were nearly identical (Fig. 2A). When the CC-I group was split into the CC-MON and CC-SWI groups, the KMA curves of both groups decreased in parallel with a steeper slope in the CC-SWI group (Fig. 3A).

**Fig. 4:**
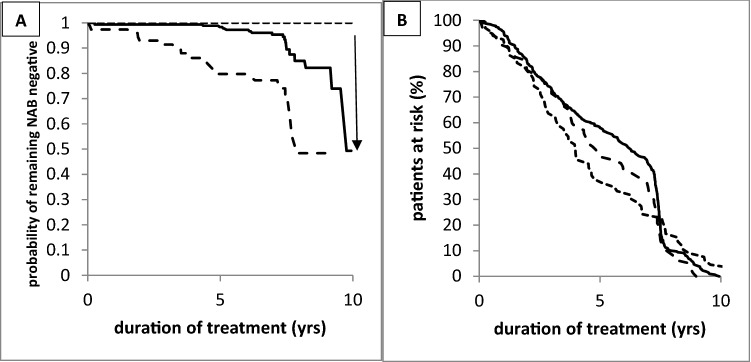
**A** Kaplan–Meier analysis of remaining MHDA-negative in the CF-MON group (light hatched line), CC-MON-10 group (full line), and CC-SWI-10 group (heavy hatched line). The arrow indicates a significant difference (*p* < 0.035). **B** Temporal development of the patients at risk in the CF-MON group (light hatched line), CC-MON-10 group (full line), and CC-SWI-10 group (heavy hatched line) is similar in all three patient groups

When the KMA curves of the CF-MON and CC-MON-10 groups were compared (Fig. 4A), the KMA curve of the CC-MON-10 group started to decrease after 5 years and became significantly (*p* < 0.035) different from the KMA curve of the CF-MON group when a 10-years period of time was analyzed. The KMA curve of the CC-SWI-10 group showed an even steeper decrease than the KMA curve of the CC-MON-10 group. During 10 years, the number of patients at risk to develop NABs continuously decreased in all three patient groups down to zero.

## Discussion

This is the first comparative study demonstrating a statistically significant difference in antigenicity between the different botulinum neurotoxin type A preparations.

### General remarks on the comparison of different studies on antibody formation

In general, studies on antibody formation following BoNT/A treatment report low antibody formation rates. However, in contrast to prevalence and incidence, the antibody rate (% of NAB-positive patients in a cohort) is not a well-defined statistical term. In many studies reporting NAB rates, only a limited number of selected patients are tested for the presence of NABs (for a review see [24]). Furthermore, antibody rates do not account for unequal durations of treatment and drop-out rates. However, for the determination of NAB prevalence, by definition, all patients of a cohort have to be analyzed. Therefore, most NAB rates reported, grossly underestimate the prevalence of NABs. The antibody rate is equal to NAB prevalence only when all patients were tested and treated over an equally long period of time. The comparison and interpretation of reported NAB rates may become even more difficult when patients are treated with varying BoNT/A doses per session and different injection patterns and are analyzed with different NAB detection tests with different sensitivities [11, 24]. Furthermore, since many studies reporting NAB rates have a mean duration of less than 3 years, these NAB rates estimate the incidence rather than the long-term prevalence of NABs [20, 24].

For a reliable measure of antibody prevalence, all members of a cohort should be tested for the presence of antibodies by means of the same assay, and a Kaplan–Meier survival analysis should be performed to estimate the probability of patients remaining antibody negative. This approach takes into account the duration of treatment and the drop-out rates at each time point. Furthermore, in comparison studies, the temporal development of the censoring process and the numbers of patients at risk for the development of NABs should be comparable over an equally long period of time [11].

### Significant differences in NAB prevalence among different treatment groups detected by MHDA assay

In the present study, ELISA screening followed by an MHDA confirmation test was used for all patients which is more sensitive for detecting relevant NAB titers than the mouse lethality assay (MLA), also referred to as the mouse protection assay (MPA) [14, 23, 25]. The statistical analysis was restricted to those patients whose BoNT/A therapy had not been initiated before 1/2005 or more than 10 years before recruitment. Thus, on the one hand, no patient who might have received the original onaBoNT/A formulation (e.g., “old” Botox^®^) with a much higher protein content and antigenicity [10, 16] was enrolled in the study. On the other hand, the numbers of patients at risk developed similarly in the CF-MON- and CC-MON-10-groups (see Fig. 4B).

None of the patients in the CF-MON group tested positive by means of the MHDA, in full agreement with another recently published study [26]. Most of the patients of that study are included in the CF-MON group. In contrast, the probability of remaining NAB-negative steeply declined with duration of treatment in the CCI-group (Fig. 2A).

Whenever a patient developed clinical hints of a PSTF, he/she was switched to incoBoNT/A. This was done, because PSTF is associated with an MHDA-positive test in up to 50% of the patients [25] and because it is our experience that NAB titers may decline, even below the detection limit of the MHDA, when patients were switched to incoBoNT/A [27].

Our selection process to detect a beginning PSTF and to switch these patients to incoBoNT/A had obviously been highly effective, since the prevalence of NABs was high (> 33%) in the CC-SWI group and low (5.9%) in the group of patients who remained on abo- or onaBoNT/A (CC-MON group). Because of this selection bias and the significantly higher mean dose in the CF-MON group, which is a relevant risk factor for NAB formation, the present statistical procedure to compare the CF-MON and CC-MON-10 groups is highly conservative and favors the CC-MON-10 group. Nevertheless, the difference in the prevalence of NABs was significantly (*p* < 0.035) lower in the CF-MON group than in the CC-MON-10 group.

### Clinical implications of the low antigenicity of incoBoNT/A

The results of this study suggest that initiation of BoNT/A therapy with incoBoNT/A monotherapy reduces the risk of NAB induction from the start of treatment [26]. In a previous study, we demonstrated that patients who develop a clinically relevant PSTF in the course of treatment may have an early reduction of efficacy, even after the first injection [28]. NAB testing in CD patients who had received less than nine abo- or onaBoNT/A injections and were then switched to long-term incoBoNT/A therapy demonstrated that the risk of becoming MHDA-positive is no longer zero after a few complex protein-containing BoNT/A injections [26]. Therefore, early induction of NABs should be considered also with awareness for the fact that each patient is initiating a potentially lifelong course of BoNT therapy.

Our data demonstrate that incoBoNT/A treatment is not associated with a significant risk of NAB induction, and previous studies suggest that NAB-positive patients switched from CC-BoNT/A to incoBoNT/A have a realistic chance that antibody titres are not further boostered and may decline over time, preserving a partial, in some cases even increasing clinical response [26, 27]. This could be essential for individual patients, especially with an increasing use of BoNT/A in clinical practice due to an increasing number of indications.

In clinical practice, the long-term outcome is relevant which has not been presented for the different treatment groups in the present study. In still-responding patients with cervical dystonia (CD) NABs may be present despite of an excellent clinical outcome [20]. This is in line with a recent study which did not detect a significant correlation between paralysis time and TSUI-score neither in MHDA-positive nor in MHDA-negative patients with CD [29]. On the other hand, NAB-positive CD patients are treated with significantly higher BoNT/A doses compared to NAB-negative CD patients and have a significant worse outcome [20, 30]. Thus, the presence of NABs is one factor for a reduced clinical response in BoNT therapy, but not the only one, as emphasized repeatedly [25, 31].

A recent study demonstrated that higher doses of incoBoNT/A [32] and shorter reinjection intervals are safe and can lead to a better patient satisfaction, which will possibly result in a higher adherence to therapy [33]. These insights, along with our finding of significant differences in antigenicity, may have a significant impact on patient management strategies in BoNT/A therapy.

The lesson learned from the transition from “old” to the “new” Botox® was that booster injections, short reinjection intervals, and unnecessary high doses per session should be avoided [10], and that the BoNT/A formulation with the lowest protein load should be used [5]. In principle, these recommendations still hold, and the present demonstration that there are differences regarding the immunogenicity among the currently licensed BoNT/A preparations is in line with these recommendations. The use of incoBoNT/A may allow more flexibility in daily clinical practice. However, more studies are warranted to analyze whether the use of higher doses of incoBoNT/A [32] and shorter reinjection intervals does not go along with a relevant increase of the risk to induce NABs and PSTF.

### Limitations of the study

We present a monocentric, cross-sectional study with a long recruitment period. Patients were not randomized, and the distribution of disease entities was slightly different in the CF-MON and CC-MON-10 groups because of the different license situations of the different BoNT/A preparations. The study is highly conservative, since those who switched preparations were thoroughly excluded from the final analysis.

A controlled longitudinal multicenter study with continuous assessment of NAB induction, clinical outcome, development of PSTF, and drop-out rates is warranted to investigate differences in probability to develop a secondary non-response and in antigenicity between different BoNT/A preparations in more detail.

## References

[1] Frevert J (2015). Pharmaceutical, biological, and clinical properties of botulinum neurotoxin type A products. Drugs R D.

[2] International Society of Aesthetic Plastic Surgeons. ISAPS international survey on aesthetic/cosmetic procedures performed in 2013. http://www.isaps.org/news/isaps-global-statistics

[3] Jost WH, Friedman A, Michel O (2019). SIAXI: placebo-controlled, randomized, double-blind study of incobotulinumtoxinA for sialorrhea. Neurology.

[4] Bellows S, Jankovic J (2019). Immunogenicity associated with botulinum toxin treatment. Toxins.

[5] Aoki KR, Guyer B (2001). Botulinum toxin type A and other botulinum toxin serotypes: a comparative review of biochemical and pharmacological actions. Eur J Neurol.

[6] Atassi MZ, Dolimbek BZ, Jankovic J, Steward LE, Aoki KR (2011). Regions of botulinum neurotoxin A light chain recognized by human anti-toxin antibodies from cervical dystonia patients immunoresistant to toxin treatment. The antigenic structure of the active toxin recognized by human antibodies. Immunobiology.

[7] Dressler D (2004). Clinical presentation and management of antibody-induced failure of botulinum toxin therapy. Mov Disord.

[8] Hefter H, Rosenthal D, Bigalke H, Moll M (2019). Clinical relevance of neutralizing antibodies in botulinum toxin long-term treated still-responding patients with cervical dystonia. Ther Adv Neurol Disord.

[9] Simpson DM, Hallett M, Ashman EJ, Comella CL, Green MW, Gronseth GS, Armstrong MJ, Gloss D, Potrebic S, Jankovic J (2016). Practice guideline update summary: botulinum neurotoxin for the treatment of blepharospasm, cervical dystonia, adult spasticity, and headache: report of the guideline development subcommittee of the American Academy of Neurology. Neurology.

[10] Greene P, Fahn S, Diamond B (1994). Development of resistance to botulinum toxin type A in patients with torticollis. Mov Disord.

[11] Albrecht P, Jansen A, Lee JI (2019). High prevalence of neutralizing antibodies after long-term botulinum neurotoxin therapy. Neurology.

[12] Frevert J (2010). Content of botulinum neurotoxin in Botox^®^/Vistabel^®^, Dysport^®^/Azzalure^®^, and Xeomin^®^/Bocouture^®^. Drugs R D.

[13] Gu S, Rumpel S, Zhou J (2012). Botulinum neurotoxin is shielded by NTNHA in an interlocked complex. Science.

[14] Frevert J, Dressler D (2010). Complexing proteins in botulinum toxin type A drugs: a help or a hindrance?. Biologics.

[15] Tsui JK, Eisen A, Stoessl AJ, Calne S, Calne DB (1986). Double-blind study of botulinum toxin in spasmodic torticollis. Lancet.

[16] Jankovic J, Vuong KD, Ahsan J (2003). Comparison of efficacy and immunogenicity of original versus current botulinum toxin in cervical dystonia. Neurology.

[17] Brin MF, Comella CL, Jankovic J, Lai F, Naumann M (2008). Long-term treatment with botulinum toxin type A in cervical dystonia has low immunogenicity by mouse protection assay. Mov Disord.

[18] Panjwani N, O'Keeffe R, Pickett A (2008). Biochemical, functional and potency characteristics of type A botulinum toxin in clinical use. Botulinum J.

[19] Blümel J, Frevert J, Schwaier A (2006). Comparative antigenicity of three preparations of botulinum neurotoxin type A in the rabbit. Neurotox Res.

[20] Hefter H, Rosenthal D, Moll M (2016). High botulinum toxin-neutralizing antibody prevalence under long-term cervical dystonia treatment. Mov Disord Clin Pract.

[21] Hefter H, Jansen A, Moll M, Ringelstein M, Albrecht P (2016). High prevalence of neutralizing antibodies in BoNT/A long-term–treated patients with focal dystonia and spasticity. Toxicon.

[22] Contarino MF, Van Den Dool J, Balash Y (2017). Clinical practice: evidence-based recommendations for the treatment of cervical dystonia with botulinum toxin. Front Neurol.

[23] Göschel H, Wohlfarth K, Frevert J, Dengler R, Bigalke H (1997). Botulinum A toxin therapy: neutralizing and nonneutralizing antibodies-therapeutic consequences. Exp Neurol.

[24] Fabbri M, Leodori G, Fernandes RM (2016). Neutralizing antibody and botulinum toxin therapy: a systematic review and meta-analysis. Neurotox Res.

[25] Lange O, Bigalke H, Dengler R, Wegner F, deGroot M, Wohlfarth K (2009). Neutralizing antibodies and secondary therapy failure after treatment with botulinum toxin type A: much ado about nothing?. Clin Neuropharmacol.

[26] Hefter H, Brauns R, Ürer B, Rosenthal D, Albrecht P (2020). Effective long-term treatment with incobotulinumtoxin (Xeomin^®^) without neutralizing antibody induction: a monocentric, cross-sectional study. J Neurol.

[27] Hefter H, Hartmann C, Kahlen U, Moll M, Bigalke H (2012). Prospective analysis of neutralizing antibody titres in secondary non-responders under continuous treatment with a botulinumtoxin type A preparation free of complexing proteins–a single cohort 4-year follow-up study. BMJ Open.

[28] Hefter H, Spiess C, Rosenthal D (2014). Very early reduction in efficacy of botulinum toxin therapy for cervical dystonia in patients with subsequent secondary treatment failure: a retrospective analysis. J Neural Transm (Vienna).

[29] Hefter H, Ürer B, Brauns R, Rosenthal D, Meuth SG, Lee JI, Albrecht P, Samadzadeh S (2022). The complex relationship between antibody titers and clinical outcome in botulinum toxin type A long-term treated patients with cervical dystonia. J Neurol.

[30] Samadzadeh S, Ürer B, Brauns R (2020). Clinical implications of difference in antigenicity of different botulinum neurotoxin type A preparations: clinical take-home messages from our research pool and literature. Toxins.

[31] Ferreira JJ, Colosimo C, Bhidayasiri R, Marti MJ, Maisonobe P, Om S (2015). Factors influencing secondary non-response to botulinum toxin type A injections in cervical dystonia. Parkinsonism Relat Disord.

[32] Wissel J, Bensmail D, Ferreira JJ (2017). Safety and efficacy of incobotulinumtoxinA doses up to 800 U in limb spasticity: the TOWER study. Neurology.

[33] Sethi KD, Rodriguez R, Olayinka B (2012). Satisfaction with botulinum toxin treatment: a cross-sectional survey of patients with cervical dystonia. J Med Econ.

